# Factors Associated with Obstetric Violence Implicated in the Development of Postpartum Depression and Post-Traumatic Stress Disorder: A Systematic Review

**DOI:** 10.3390/nursrep13040130

**Published:** 2023-11-01

**Authors:** Claudia Susana Silva-Fernandez, Maria de la Calle, Silvia M. Arribas, Eva Garrosa, David Ramiro-Cortijo

**Affiliations:** 1 Department of Biological & Health Psychology, Faculty of Psychology, Universidad Autónoma de Madrid, C/Ivan Pavlov 6, 28049 Madrid, Spaineva.garrosa@uam.es (E.G.); 2 Obstetric and Gynecology Service, Hospital Universitario La Paz, Universidad Autónoma de Madrid, Paseo de la Castellana 261, 28046 Madrid, Spain; 3 Department of Physiology, Faculty of Medicine, Universidad Autónoma de Madrid, C/Arzobispo Morcillo 2, 28029 Madrid, Spain; 4 Instituto Universitario de Estudios de la Mujer (IUEM), Universidad Autónoma de Madrid, C/Francisco Tomás y Valiente 5, 28049 Madrid, Spain

**Keywords:** postpartum depression, post-traumatic stress disorder, maternal mental health, women’s health, obstetric violence, obstetric vulnerability

## Abstract

Postpartum depression (PPD) and post-traumatic stress disorder (PTSD) continue to be prevalent, and disabling women with mental disorders and obstetric violence (OV) may be a trigger for them, particularly during maternity. We aimed to analyze the association between manifestations of OV with the development of PPD and PTSD during pregnancy, childbirth, and postpartum. This systematic review was based on the PRISMA 2020 statement and explored original articles published between 2012 and 2022. A total of 21 articles were included in the analysis, and bias was assessed by the Effective Public Health Practice Project’s Quality Assessment Tool. The highest rate of PPD symptoms appeared in women under 20 years old, multiparous, and with low education levels. The higher PTSD ratio was present in women under 35 years, primiparous, and with secondary studies. The mode of labor (instrumental or C-section) was identified as a major risk factor of PPD, being mediator variables of the informal coercion of health professionals and dissatisfaction with newborn healthcare. Instead, partner support during labor and high satisfaction with healthcare during birth were protective factors. Regarding PTSD, the mode of labor, several perineal tears, and the Kristeller technique were risk factors, and loss of autonomy and coercion modulated PTSD symptomatology. The protective factors for PTSD were respect for the labor plan, adequate communication with health professionals, social support during labor, and the skin-to-skin procedure. This systematic review provides evidence that OV contributes to PPD and PTSD, being important in developing standardized tools to prevent it. This study recommends changes in maternal healthcare policies, such as individualized healthcare assistance, humanized pregnancy protocols, and women’s mental health follow-up, and improvements in the methodological quality of future research.

## 1. Introduction

In 2014, the World Health Organization (WHO) recognized obstetric violence or vulnerability (OV) due to healthcare practices as a global issue with high social and health consequences that must be prevented [1]. OV was defined as breaking women’s healthcare rights due to attitudes, actions, or omission of health practices during pregnancy, childbirth, or postpartum [2]. Among the countries with the highest OV rate are Ethiopia (75.1%) [3], Colombia (69.0%) [4], Mexico (33.3%) [5], and Venezuela (26.3%) [6]. In Spain, it was estimated at 67.4% [7].

It has been declared that the developing contexts linked to OV can contribute to developing mental and physical difficulties such as postpartum depression (PPD) and post-traumatic stress disorder (PTSD) [8,9]. PPD is one of the major complications during postpartum, with an estimated prevalence between 17% and 47% [10,11]. This wide variability in PPD prevalences has been explained by the differences in the use of non-validated instruments, cultural variables, socio-economic environments, perception of mental health, and biological vulnerability factors [12]. Socio-economic deprivation, low educational attainment, fall in self-esteem and self-efficacy, insecure attachment style in childhood, history of violence and depressive somatic symptoms, weak social support, unplanned pregnancy, and poor prenatal care have been identified as risk factors for PPD [13,14,15,16]. PTSD is rising during the postpartum period [17], with a prevalence of 4.7% [18] and up to 15.7% in risk groups (i.e., women with past trauma and psychological issues, low social support, and/or traumatic birth experience) [19]. Common risk factors associated with PTSD are fear of childbirth, insecure attachment style in infancy [9], a premature newborn with low birth weight, low educational level [20], psychological disorders, and surgical interventions [21].

PPD and PTSD are characterized by a refusal attitude for life and ambivalent behavioral, cognitive, and somatic disorders. In addition to the social and healthcare costs, both mental difficulties entail negative effects on the physical and emotional health of the woman, leading to maladaptation to motherhood and serious misalignments in the affective and physical development of the newborn [22,23,24]. It has been identified that PPD and PTSD can be activated one year after childbirth [18,23].

Therefore, this article intends to gain knowledge on the impact that OV has on maternal health, particularly on PPD and PTSD. This would be a critical aspect to promote women’s health during maternity. The aim of this systematic review is to explore the risk and protective factors associated with OV that predispose to the development of PPD and PTSD during pregnancy, childbirth, and postpartum. This will help to design health policies and strategies focused on women’s mental health.

## 2. Materials and Methods

### 2.1. Study Design

The present study is a systematic review following the PRISMA 2020 guidelines (Preferred Reporting Items for Systematic Reviews and Meta-Analyses), according to Matthew et al. [25]. The PICO strategy of this review proposes to determine the scientific findings on the association between OV and risk factors for PPD or PTSD in postpartum women. Thus, it will be extracted the key variables of this systematic review and its operationalization.

This review considered PPD as a mental disorder whose main symptoms would be depressed mood, guilt, feeling worthless, difficulty concentrating, excessive worry, sleep disorder, or weight changes, which can occur from the last month of pregnancy to one year after delivery [26]. PTSD was defined as a mental disorder developed from perception or exposure to a potentially traumatic event, whose symptoms are reliving this stressful event, feeling of disconnection with the newborn, absence of reality, nightmares, irritability, rejection of motherhood, tokophobia, increased arousal, fear, and concentration problems [27]. The units of analysis included studies in which the target was the OV during pregnancy, birth, or postpartum as a risk factor or mediator for PPD and PTSD.

### 2.2. Search Strategy of the Studies

The search was carried out in the databases APA, Medline, PubMed, Cochrane Library, ProQuest, CINAHL, Dialnet, Scopus, Psicodoc, Scielo, Google Scholar, Web of Science, and Hinari. Gray literature was not consulted. The strategy was applied using free and MeSH terms in the abstract by the combinations of keywords of the variables, using booleans AND, OR, and NOT.

The free terms were: “obstetric violence” (“obstetric violence” OR “medical mistreatment” OR “medical negligence” OR “medical abuse” OR “medical disrespect”), AND “maternity” (“maternal” OR “pregnancy” OR “birth” OR “postpartum”) AND “disorder postpartum” (“major depressive disorder” OR “post-traumatic stress disorder”) AND “psychosocial factors” (“psychosocial factors” OR “risk factors” OR “predictors”).

The MeSH terms were depression postpartum OR stress post-traumatic AND gender-based violence OR malpractice OR physical abuse OR emotional abuse, NOT domestic violence AND risk factors AND pregnancy OR postpartum period OR parturition. The reference list provided in the selected studies included in this review was also consulted to identify other potential documents to explore.

The inclusion criteria were research articles published in peer-reviewed journals between 2012 and 2022 in Spanish, English, and Portuguese languages, which answered the PICO question of this review. Subsequently, articles were excluded if there were non-full access, duplicates in the consulted databases, reviews, meta-analyses, or single-case studies.

### 2.3. Selection Process of the Studies

**Figure 1:**
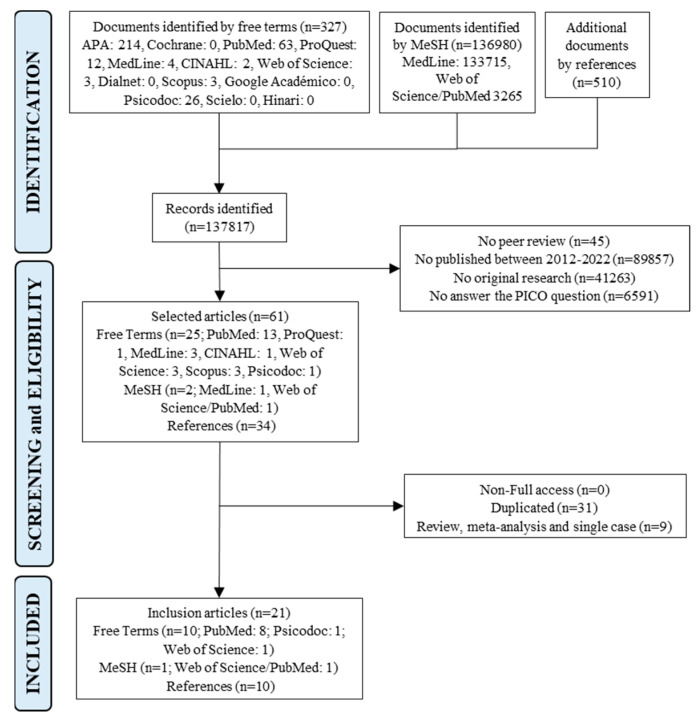
Flow chart of the selection process of the analysis units according to the PRISMA guidelines 2020; MeSH, medical subject headings; n, number of analysis.

Firstly, a total of 137,817 documents were obtained (137,307 corresponding to databases and 510 from the references of the selected studies), which were filtered according to inclusion criteria, identifying 61 articles. These documents were carefully analyzed, reading the title and abstract. Secondly, articles that evaluated PPD or PTSD together with OV actions (i.e., episiotomy, the performance of the Kristeller technique or fundal uterine pressure maneuver, disrespect for the labor plan, rejection of social support, discrimination, among others) were incorporated, and non-full access, duplicated reviews, meta-analysis, and single cases studies were excluded. The articles that analyzed the satisfaction between healthcare of professionals and maternity were explored. Finally, a total of 21 articles were selected for this review: 10 using free terms, 1 using MeSH, and 10 derived from the reference list of the selected articles (Figure 1).

### 2.4. Data Collection

The included articles were exhaustive and analytically reviewed, extracting the publication data such as authors, year, journal, country, expertise field, and methodological aspects (study design, sample size, and instruments used). The publication area was plotted using the *mapdata* package by R software (version 4.3.1; R Core Team 2022. R Foundation for Statistical Computing, Vienna, Austria; https://www.R-project.org/ accessed on 9 September 2023) with RStudio interface (version 2023.06.0 + 421 for Windows; Boston, MA; USA). The definition of variables and diagnostics (including questionnaires and used tools), main outcomes (including sociodemographic characteristics and prevalence of OV, PPD, PTSD), and factors (considering adjusted odd ratio and standardized coefficient by the regression models) were extracted. All regression coefficients were expressed with a 95% confidence interval. In addition, the recommendations from clinical practice and future research goals were also collected.

The factors to identify OV were classified in three dimensions: (1) medical interventions that are painful or disabling and could be avoided under clinical supervision (episiotomy, amniotomy, Kristeller technique, C-section, instrumental labor, perineal tears, use of the synthetic oxytocin, manual removal of the placenta and birth injury); (2) actions of the professional healthcare (mistreatment, discrimination, offensive and coercive communication, disrespect to labor plan and privacy, poor clarity in the information, minimize women’s autonomy, bullying, refusal of pain relief, deprive women to express emotions or questions, skin-to-skin with infant, breastfeeding in the first hour after delivery and social support); and (3) perception of dissatisfaction with healthcare during childbirth due to failure to meet expectations and needs.

Data were collected in a database designed by the research team using the online “Critical Appraisal Tools (FLC 3.0)” developed by the Basque Office for Health Technology Assessment (OSTEBA) [28], according to a previous publication [29].

### 2.5. Evaluation of the Quality of the Studies

The Effective Public Health Practice Project from Canada developed the Effective Public Health Practice Project Quality Assessment Tool (EPHPP; [30]), which was used as a generic tool to evaluate a variety of intervention study designs. This tool has been judged suitable to be used in systematic reviews of effectiveness [31]. Under EPHPP, the Qualitative Assessment Tool for Quantitative Studies was applied to analyze the bias of each article and to determine the level of confidence according to the categorical scale. This tool was already implemented as a methodological strategy in systematic reviews [30]. This instrument explores the overall bias range of studies based on six domains: selection (2 items), design (4 items), confounding factors (2 items), blinding (2 items), data collection methods (2 items), and withdrawals or drop-outs (2 items). Guidelines for this tool indicate that each domain is rated as strong (3 points), moderate (2 points), or weak (1 point), and domains are averaged to provide the total score. The maximum score is 3, and each study is assigned a quality rating of low risk of bias (1.0–1.5), moderate/unclear concerns of bias (1.51–2.5), or high risk of bias (2.51–3.0). Additionally, the qualitative range was plotted using the *robvis* package [32] by R software with the RStudio interface.

## 3. Results

### 3.1. Demographic Analysis of the Studies

**Figure 2:**
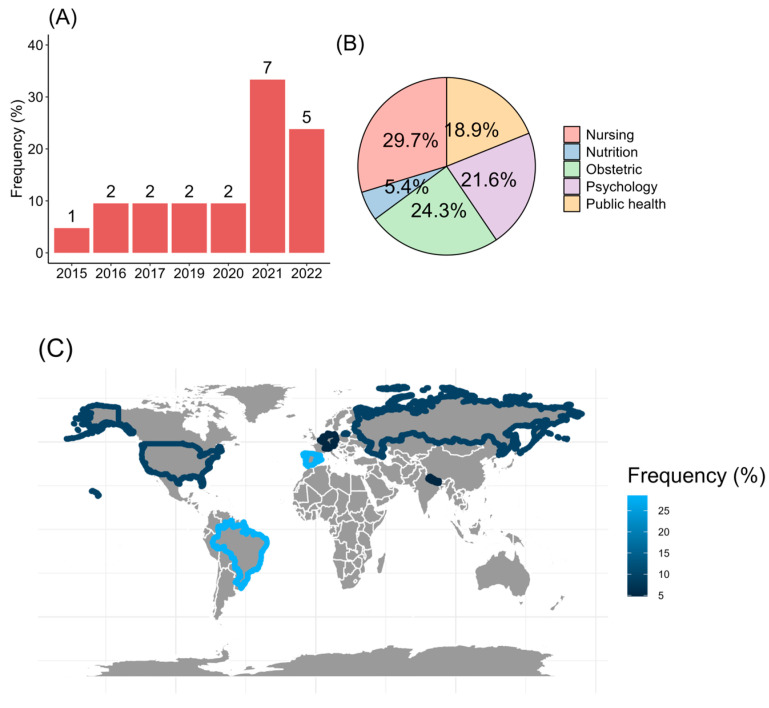
Screening of the articles regarding (**A**) the year of the publication, (**B**) expertise of knowledge, and (**C**) worldwide allocation.

The present review found 21 studies that matched inclusion and exclusion criteria and were published between 2015 and 2022. The major rate of publication was found in 2021 (33.3%, 7/21; Figure 2A), with most of the articles published in English (95.2%, 20/21). The field expertise was in health sciences, specifically medicine, nursing, and psychology (Figure 2B). In addition, the studies were carried out in Europe, with 28.6% (6/21) from Spain. From America, Brazil reported 28.6%, and the United States added 9.5% of the studies. From Asia, it was found that 4.8% (1/21) from Nepal (Figure 2C). 

### 3.2. Quality Analysis of the Studies

**Figure 3:**
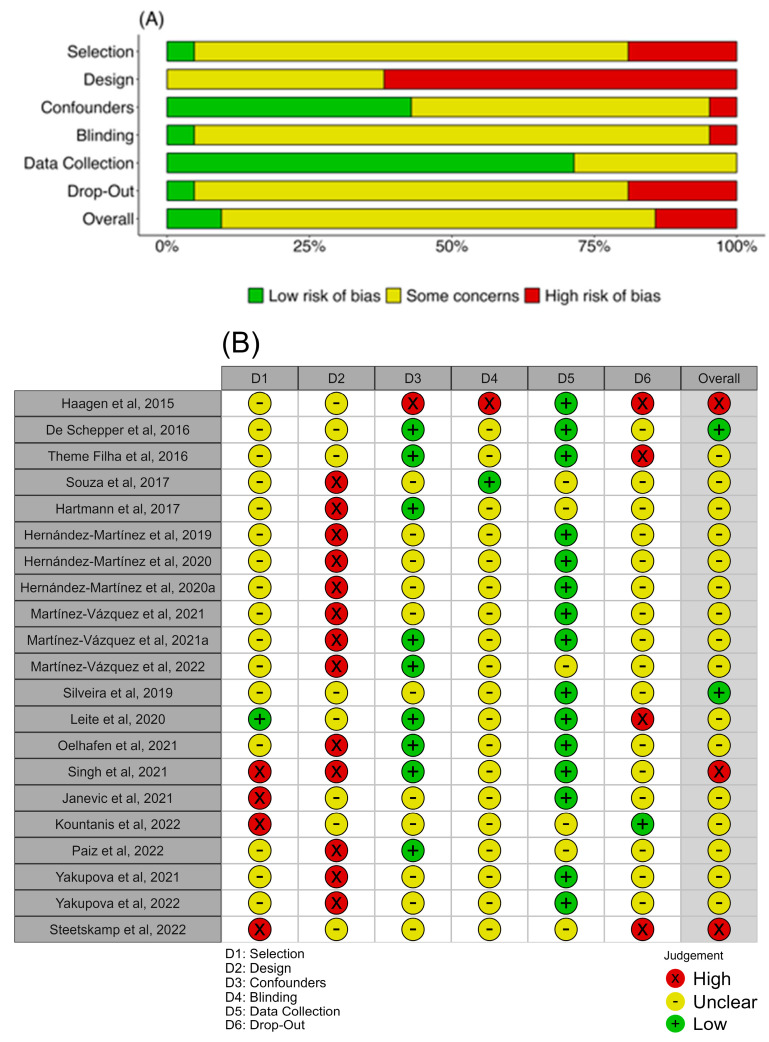
Risk-of-bias assessment by the Qualitative Assessment Tool for Quantitative Studies. (**A**) Weighted bar plot shows the proportion of biased judgment separately for each domain in the assessment tool. (**B**) The traffic light plot with every judgment in a matrix, with the domain of the tool (D1–6) and each study down the vertical [33,34,35,36,37,38,39,40,41,42,43,44,45,46,47,48,49,50,51,52,53].

The articles showed a high proportion of “some concerns” in all analyzed domains, with a high risk of bias in the study design domain and low risk in the data collection domain since several tools showed significant reliability coefficients and validity (Figure 3A). The selection blinding and drop-out identification were the domains that showed unclear bias in most of the analyzed articles (Figure 3B).

### 3.3. Postpartum Depression and Post-Traumatic Stress Disorder Outcomes

Regarding PTSD, 10 articles were found [33,34,35,36,37,38,39,40,41,42]. With respect to PPD, nine articles were found [43,44,45,46,47,48,49,50,51]. In addition, in two studies [52,53], PTSD and PPD were simultaneously analyzed. Regarding the instruments used, the Edinburgh Postnatal Depression Scale (EPDS) predominated in identifying symptoms of PPD, using a cut-off ≥ 10 (Table 1), and the Perinatal Post-Traumatic Stress Disorder Questionnaire (PPQ) was the most used tool for PTSD with a cut-off ≥ 19 (Table 2). Despite these findings, the variability in the cut-off used between investigations was notorious.

In the analysis of the methodology, it was identified that most of the studies correspond to a cross-sectional method (14/21, 66.6%) [35,36,37,38,39,42,44,45,48,49,50,51,52,53], and their sample size ranged from 112 to 23,894 women, all in the postpartum period.

In the studies analyzed, the prevalence of PPD was between 5.7% and 45.7%. The mean age of women with PPD symptoms was 25.6 years. The highest rate of symptoms compatible with PPD or elevated EPDS scores were in women under 20 years, multiparous (more than one previous child), and women with a low level of education (illiterate or primary studies).

Considering the relevance of the first 6 months of postpartum to the development of PPD, 80.9% (17/21) of the articles explored this period [33,34,35,38,40,41,42,43,44,45,46,47,48,49,50,51,53]. Other articles evaluated the period between 7 and 12 months postpartum (9/21, 42.8%) [33,36,38,39,40,43,47,48,51]; 14.2% (3/21) studied from 13 months to 5 years postpartum [36,39,52]; and one article did not specify the period evaluated [37].

The prevalence of PTSD was between 0.3% and 24.5%. The studies usually classify women as under or over 35 years old, 32.5 years being the mean age of women with PTSD symptoms. The women who presented higher PTSD ratios were women under 35 years, primiparous (non-previous labor) being heterogeneous in educational level, and the majority with secondary studies, such as a high-school degree.

### 3.4. Obstetric Violence Explored in the Articles

To explore OV, ad hoc tools were preferred, although the association between OV with quality and humanization of the clinical practices was assessed by the Birth Satisfaction Scale [53], the 11-item satisfaction scale [49], and Salmon’s Item List [48]. All studies analyzed the actions of OV during labor, birth, and immediate postpartum care, and only one of the studies explored OV in pregnancy and postpartum [37].

When the studies used a general measure of OV, 22.6% to 45.2% perception of OV was detected [47,52]. About the type, physical OV was detected in 1.2% to 59% [38,39,44,46,51,52], verbal OV was detected in 9% to 50.3% [38,39,44,46,51,52], and psycho-affective OV was detected in 25.4% to 35.2% [38,39,51]. In the conceptualization of the key variables, it was evident that 57.1% of the studies did not define OV since it was not associated with their main study outcome.

PPD and PTSD were considered common mental disorders in postpartum, which can develop until one year after labor, with negative consequences for the woman and her infant. PTSD was always associated with traumatic experiences, such as childbirth and painful medical interventions. PPD was globally the main postpartum complication. The definitions of these mental disorders were mainly related to symptoms in the emotional, cognitive, and behavioral components.

### 3.5. Factors Associated with Obstetric Violence and Contribution to Develop PPD and PTSD

The 14.2% (3/21) of the studies classified OV into physical, verbal, and psycho-affective violence [38,39,51]. Other mentioned factors identified by women as OV were dissatisfaction about her/newborn healthcare, social support restriction, not following the labor plan or not agreeing on the induction of labor, not advising on procedures/techniques during delivery and postpartum care, and miscommunication with medical staff.

The rate of dissatisfaction with her or her newborn healthcare was between 1.7% and 73.1% [37,43,45,49,50,53]. The medical interventions associated with OV were C-section (6.6% to 51.9%) [34,36,38,39,41,42,43,48,51,52], labor induction (12.1% to 41.4%) [33,34,36,37,38,39,42,51,52], episiotomy (9.5% to 41.4%) [34,35,36,37,38,39,42,51,52], and Kristeller technique (3.1% to 30.8%) [35,42,52].

The most common negative actions from the medical staff for OV were unfollow the birth plan (13.3% to 79.4%) [35,36,37,38,39], deprivation of social support during delivery (1.9% to 73.0%) [41,43,51,52], disrespectful and discriminatory treatment (0.9% to 65.5%) [39,42,46,48,49,52,53], coercive communication or poor information (6.2% to 40.9%) [40,42,48,49,50,52], and to avoid skin-to-skin contact (20.7% to 35.5%) [35,36,37,39,50,51].

The mode of labor (instrumental and/or C-section) was identified as a major risk factor for PPD [48,49]. Therefore, informal coercion of health professionals [48] and dissatisfaction with newborn healthcare [49] were mediators for symptoms of PPD. In addition, not allowing social support increases disrespect and abuse during labor, which is also associated with higher rates of PPD [47]. Protective factors of PPD were to allow partner support during labor [44] and high satisfaction of healthcare during birth [45,53] (Table 3).

The forms of OV considered by the women related to the development of PTSD were the mode of delivery (instrumental and C-section) [33,35,36,37,38,39,41], several perineal tears [35,36,37,39], Kristeller technique [35,36], and labor induction [39]. Also, loss of autonomy and coercion contributed to increased symptoms of PTSD [38,39] while respecting the labor plan, locus of control and communication [33,34,35,36], allowing social support during labor [41], and the skin-to-skin procedure [36,37] were protective factors for PTSD. When the woman had a positive perception of her health assistance and respectful communication during healthcare, the PTSD symptomatology was reduced [39,40,53] (Table 4).

During maternity, disrespect, abuse [46,47], and mistreatment [50] were associated with PPD and PTSD. When the studies evaluated the type of OV, physical [39,44,46], verbal [38,39,44,46,51], and psycho-affective violence [38,51] were risk factors for developing PPD and PTSD. In addition, negligence was a risk factor for developing PPD [44]. The violence experienced during labor and birth was a significant predictor of PTSD and PPD [38,39,42,44,46,52]. The perception of respect of health professionals was a protective factor of PTSD [37].

## 4. Discussion

WHO evidences the importance of investigating and preventing the complications of violation of women’s rights during pregnancy, childbirth, and postpartum care [1,54,55]. In addition, there is growing interest in the study of OV and its effects on women’s mental health, as shown by the increased number of publications on this subject and interdisciplinary fields. However, the progress is still incipient because few studies directly analyze OV, limited studies explore OV during postpartum and not only during pregnancy, and there is a variety of methodologies.

According to the reviewed studies, the OV definition can be established as a failure in women’s rights during labor and birth healthcare. OV can be manifested as disrespect, limitation of a woman’s autonomy or coercion, misinformation, lack of privacy, physical, sexual, or verbal abuse, psycho-affective abuses, discrimination, neglect of childcare, and forbidding the newborn–mother interaction, associated with unsatisfied expectations of the women [38,39,41,46,47,48,50,51]. This definition covers the actions that disrupt human rights during labor healthcare assistance but does not include pregnancy and postpartum [56] periods of vulnerability associated with physical, psychological, and social changes for the mother [8,41].

It should be noted that the definition of OV is complex due to the connotation of the term “violence”. Violence implicates the maltreatment or intention to harm with a malicious legal meaning. However, in some cases, health practice can be confused with failure to meet expectations related to pregnancy or childbirth without implying intent to harm by healthcare providers. Therefore, we believe that for the greater satisfaction of the women, the health services should provide humanized protocols that control excessive and misinformed interventions during pregnancy, labor, and postpartum. Training in the gender field prevents negative consequences on women’s mental health. These actions will promote good clinical practices and improve protocols that, for the moment, could be based on old structures in which the integral well-being of the pregnant woman was neglected.

Thus, OV is a phenomenon that has not been fully recognized in health systems, demonstrated by the weakness of the definition, the difficulty in exploring healthcare satisfaction, and the lack of protocols to identify OV, showing the vulnerability of women´s rights during pregnancy, childbirth, and postpartum. Other authors have affirmed the poor knowledge of the health providers to identify the manifestations of OV [2,57] as well as its stigmatization and normalization [58,59]. In addition, this systematic review confirms the high prevalence of OV, as previously reported [5,6]. Several studies reveal that prioritizing medical interventions over women’s management control and autonomy can lead to women’s painful experience of obstetric assistance [60], affecting postpartum maternal health and dehumanization of clinical care of women [57,61]. This reinforces that OV is a health issue that needs to be recognized and intervened by professionals, the health system, governments, and society [17,62]. In this way, it will be able to humanize pregnancy and maternity. Therefore, women-centered healthcare models, where women are experts in their own bodies, could be the most effective to prevent OV.

OV is a problem by itself; in addition, it can contribute to the development of mental disorders, such as PPD and PTSD, during the postpartum, which is a sensitive and vulnerable period for women’s mental health. PPD is a frequent mental disorder with a maintained prevalence over time [10,11,15]. PDD and PTSD can be increased if associated with stressful experiences during obstetric care [17,18,19,63].

According to the characteristics of the women, it was observed that the older age, the lower PPD symptomatology. Other studies found that the age of maternity was negatively associated with depression during pregnancy [64]. Older women may lead to greater coping with childbearing, but this result may be modulated by educational level or economic stability. Women with lower educational levels and a larger family core (multiparous) had a higher ratio of PPD, which may be controlling factors in healthcare, such as perinatal psychologists. The symptomatology of PTSD was more prevalent in older women compared to PPD. Also, the risk factors were heterogeneous, being primiparous and women with high levels of education. It may be that traumatic events would be more prevalent as years are gained, and pregnancy could be an anxiety-generating condition as maternity is delayed.

Regarding the risk factors associated with OV for developing PPD and PTSD, all the analyzed studies found at least one manifestation of OV, demonstrating the negative impact of OV on women’s mental health during postpartum. Thus, OV can be evaluated by women as a traumatic experience during pregnancy, childbirth, and postpartum and associated with negative expectations and skills about motherhood [13,19,65], increasing the risk of PPD and PTSD [8,19,66]. Consistently, it has already been stated that any type of breach of rights against women has repercussions on their integral health and quality of life [57,61]. Specifically, painful and disabling interventions during labor were associated with PTSD, perceived as traumatic by women, and became a stress trigger [67]. Similarly, it occurs with the loss of autonomy. This can be explained due to the non-compliance with labor expectations (e.g., changing the established labor plan from a vaginal birth to a C-section), causing a negative perception of their labor experience [41,68]. However, although labor is a physiological event, it is also a medical activity that, thanks to its medicalization, has reduced maternal and fetal mortality, which still exists in developing countries.

In the case of PPD, the pattern of hopelessness and helplessness as negative affect is corroborated [65] since the included studies found an association with negligence, physical violence, and displeasure of newborn healthcare. These variables are related to lack of protection. Social support is a protective factor, increasing women’s maternal skills and coping in often ambivalent situations (being a mother versus the many difficulties of motherhood) [13]. Among the methodological characteristic predominance, the evaluation of PPD and PTSD in the first 6 months postpartum and few assessed during the first year. This is consistent with the clinical criteria from the first weeks to the first year postpartum or after the stressful event, which is a critical period for developing PPD and PTSD symptoms [69]. However, other studies propose evaluating after 1 year postpartum due to chronicity and the importance of the residual symptoms that can entail late activation of the disorder [70,71].

The EPDS is the most used instrument in the evaluation of PPD symptoms, which is consistent with another author [71]. However, the cut-off ≥10 can denote a selection bias, showing more cases of risk than cases of diagnosis of PPD, indicated as a limitation by the authors [45,50,52]. Therefore, researchers recommend using a cut-off ≥13 and complementing the evaluation with other instruments, such as observations and clinical interviews, to establish a diagnosis of PPD [71,72,73]. Following the appropriate cut-off, the EPDS is sensitive to identifying PPD symptoms, has been validated, and is easy to administer, proving to be a useful tool in preventive and screening women at risk [73].

The PPQ was a widely used instrument to measure PTSD, and psychometric studies indicate its viability for postpartum maternal evaluation [74,75], especially when risk experiences occurred during pregnancy and childbirth [8]. Other authors recommend the City Birth Trauma Scale (CBTS), which is updated to the diagnostic criteria established in DSM-V [67,76,77]. Furthermore, it is necessary to affirm that more validation studies are required for clinical PTSD diagnoses because the correct diagnosis is incidental in the treatment of women’s mental health for the control of the risk complications [16]. In addition, limited studies explore scales of OV. Gonzáñez-de la Torre et al. demonstrated that women perceive different degrees of OV during childbirth. These authors validated that The Obstetric Violence Scale (OVS) is useful for measuring women’s perception of OV in Spanish countries [78]. The authors explained that the design of new instruments should consider cultural context, particularly in social vulnerability environments. In the German population, Limmer et al. validated an instrument for the assessment of abuse in labor, where it was demonstrated that disrespect and abuse during childbirth contribute to PTSD symptoms [79].

### 4.1. Strengths, Limitations, and Future Perspectives

A strength of this review and considering that the OV is an incipient topic with little conceptual delimitation, a high number of included articles were identified by screening process and reference lists. This strategy is effective to deep and was not limited to the exploration-free terms and MeSH. However, the heterogeneity in methodology, instruments used for PPD and PTSD, and characteristics of the sample would be limitations of this review. Therefore, the studies were divergent in the variables associated with OV and their methods of assessment. In turn, the findings of the review show a predominance study from Spain and Brazil, which entails recommending extending the research on OV and its association with mental health to other geographical areas that even have legal recognition of OV [2,80] and others with a high risk of OV due to cultural elements or even limited health resources [3,22].

The findings reinforce the need to adapt obstetric healthcare protocols for flexible actions focused on women [81]. At the same time, considering the risk that postpartum has on women’s mental health and the cost of quality of life and treatment for themselves and their families [18,22]. This requires the involvement of the government to modify public policies and protocols to train health professionals and strengthen health systems from the biopsychosocial model [17]. Protocols to prevent OV and humanization of pregnancy should be based on psychological and social support during all maternity processes [41,42,49,52], skin-to-skin contact [35,36,52], and the participation of women in decisions about clinical and interventions [34,35,36,46,48]. In addition, it is relevant that the health provider affords respectful and clear communication about the expectations of the woman, clinical procedures, and the potential complications and alternatives to be carried out [33,40,41,48,49]. Also, the health system should facilitate access to mental health services during pregnancy, childbirth, and postpartum [35,43,45,49,52] and monitor OV [37,50,51,52] to prevent negative effects on maternal mental health.

### 4.2. Recommendations to Prevent Obstetric Violence or Vulnerability

For health services and epidemiological studies:

- To systematically and chronologically review all obstetrical and postpartum protocols;
- To design humanized protocols based on women-centered healthcare models;
- To implement evaluation protocols for PPD and PTSD, at least at 6 months and 1 year postpartum;
- To develop OV scales adjusted by social vulnerability factors;
- To study the modulation of OV by psychosocial factors such as educational level, nationality, or gender violence.

For the healthcare professionals:

- To train in the gender field;
- To inform the women of each intervention during pregnancy, childbirth, and postpartum;
- To prepare the women for potential change that can happen during childbirth;
- To consider routine screening of PPD and PTSD reflecting maternal age, educational level, economic status, and social support;
- To establish a diagnosis of PPD and PTSD using specific scales with appropriate cut-offs, complemented with clinical and interview observations.

For the women:

- To express difficulties and emotions and request help if necessary;
- To prioritize management control and autonomy;
- To reinforce social support.

## 5. Conclusions

This systematic review of evidence on OV and its relationship with PTSD and PPD during pregnancy and postpartum and identified factors that contribute to or counteract their development. Humanization health protocols should include informing the woman of each step during labor and postpartum, do not perform excessive vaginal touching, avoiding amniorrhexis, enemas, vaginal shaving, or perineal washing, allowing the partner during labor, as well as newborn skin-to-skin and immediate breastfeeding. Furthermore, training in gender disciplines and communication skills should be central to the education of future professionals in health. This review intends to create awareness about the problem of OV and the weight of its multiple manifestations and external factors to transform the perspectives and actions of health institutions and professionals. We expect that the data synthesized in this review can promote changes in clinical protocols, supported by the evidence, and lead research to improve women’s health assistance during pregnancy and postpartum.

## Figures and Tables

**Table 1:** Characteristics of the articles studying postpartum depression.

Document	Method	Sample	Period	Tool	Criteria	Definition of OV	Measurements of OV
Theme Filha et al., 2016 [43]	Longitudinal	23,894 * 11,925 **	6 h after birth, 45 days to 6 months postpartum, and 6 to 18 months postpartum	AdHoc-SC AdHoc-OV EPDS CH	EPDS ≥ 13	Not established	INT-PD ACT-PH PER-SC
De Souza et al., 2017 [44]	Cross-sectional	432	0–3 months postpartum	AdHoc-SC AdHoc-OV EPDS-6	EPDS-6 ≥ 6	Institutional violence in birth due to failure to act or omission in healthcare	INT-PD ACT-PH
Hartmann et al., 2017 [45]	Cross-sectional	2.687	48 h postpartum	AdHoc-SC AdHoc-Ps EPDS	EPDS ≥ 10	Not established	PER-SC
Silveira et al., 2019 [46]	Longitudinal	4275 * 3065 **	Between 16 and 22 weeks of gestation. 3 months postpartum	AdHoc-SC AdHoc-OV EPDS	EPDS antenatal ≥ 10; EPDS postpartum ≥ 13	Human rights violations during childbirth associated with interactions between patient and provider of healthcare and expressed as verbal, physical, sexual abuse, discrimination, or neglect	ACT-PH
Leite et al., 2020 [47]	Longitudinal	23,378	24 h postpartum, between 43 days and 6 months postpartum, and between 6 and 12 months postpartum	AdHoc-SC AdHoc-VC EPDS CH	EPDS ≥ 13	Institutional violence against women during labor and birth. Physical, sexual, or verbal violence, stigmatization and discrimination, failure to meet standards of care	ACT-PH
Oelhafen et al., 2021 [48]	Cross-sectional	6054	0–12 months postpartum	AdHoc-OV SIL WQ	WQ = NI	Loss of autonomy due to coercion to accept obstetric interventions. Verbal violence during healthcare	ACT-PH
Singh et al., 2021 [49]	Cross-sectional	415	10 weeks postpartum	AdHoc-SC EPDS 11-IS	EPDS ≥ 10	Not established	ACT-PH PER-SC
Janevic et al., 2021 [53]	Cross-sectional	237	0–2 months postpartum	AdHoc-SC BSS-R DMS PHQ-2 C-DSM-V	PHQ-2 ≥ 3	Not established	ACT-PH PER-SC
Paiz et al., 2022 [50]	Cross-sectional	287	31–37 days postpartum	AdHoc-SC AdHoc-OV EPDS	EPDS ≥ 8	Mistreatment, disrespect, and abuse during childbirth. Gender violence by physical and psychological abuse, discrimination, neglect, lack of privacy, unconsented procedures, and poor information	ACT-PH PER-SC
Yakupova et al., 2021 [52]	Cross-sectional	611	14 months postpartum	AdHoc-SC AdHoc-OV EPDS CBTS	EPDS ≥ 10	Not established	INT-PD ACT-PH
Martínez-Vázquez et al., 2022 [51]	Cross-sectional	782	0–12 months postpartum	AdHoc-SC AdHoc-VC AdHoc-OV EPDS	EPDS ≥ 10	Violation of physical, verbal, or psycho-affective through disrespectful treatment, medical coercion, and specific clinical practices	INT-PD ACT-PH

* Finished the first assessment; ** completed the final assessment. PPD: postpartum depression. NI: was not exposed in the document. OV: obstetric violence or vulnerability due to healthcare. AdHoc-SC: ad hoc questionnaire to collect sociodemographic data. AdHoc-VC: ad hoc questionnaire for clinical variables. AdHoc-OV: ad hoc questionnaire to identify actions associated with obstetric violence or birth experience. AdHoc-Ps: ad hoc questionnaire for psychological variables (emotions, perceptions, support, behavior). EPDS: Edinburgh Postnatal Depression Scale. CH: clinic history. SIL: short version of Salmon’s Item List. WQ: Wooley Questions. 11-IS: 11-item satisfaction scale. BSS-R: Birth Satisfaction Scale—Revised. DMS: Discrimination in Medical Settings Scale. PHQ-2: Patient Health Questionnaire. C-DSM-V: DSM-V diagnostic criteria. CBTS: City Birth Trauma Scale. The measurement of OV was classified as follows: INT-PD: medical interventions that are painful or disabling and could be avoided under clinical supervision (episiotomy, amniotomy, Kristeller technique, C-section, instrumental labor, perineal tears, use of the synthetic oxytocin, manual removal of the placenta, and birth injury); ACT-PH: actions of the professional healthcare (mistreatment, discrimination, offensive and coercive communication, disrespect to labor plan and privacy, poor clarity in the information, minimize women’s autonomy, bullying, refusal of pain relief, and deprive to women of expressing their emotions or questions, skin-to-skin with infant, breastfeeding in the first hour after delivery, and social support); and PER-SC: perception about satisfaction of healthcare during childbirth based on meeting needs.

**Table 2:** Characteristics of the articles studying post-traumatic stress disorder.

Document	Method	Sample	Period	Tool	Criteria	Definition of OV	Measurements of OV
Haagen et al., 2015 [33]	Longitudinal	385 * 284 **	Pregnancy, first week postpartum, 3 months postpartum, and 10 months postpartum	AdHoc-SC AdHoc-OV PSS-SR SF-MB	PSS-SR ≥ 18	Not established	INT-PD ACT-PH
De Schepper et al., 2016 [44]	Longitudinal	340 * 229 **	1 and 6 weeks postpartum	AdHoc-SC AdHoc-VC IES-R TES	IES-R ≥ 24	Not established	INT-PD ACT-PH
Hernández-Martínez et al., 2019 [35]	Cross-sectional	2.990	4 and 6 months postpartum	AdHoc-SC AdHoc-VC PPQ	PPQ ≥ 19	Not established	INT-PD ACT-PH
Hernández-Martínez et al., 2020 [36]	Cross-sectional	1531	1 to 5 years postpartum	AdHoc-SC AdHoc-VC PPQ	PPQ ≥ 19	Not established	INT-PD ACT-PH
Hernández-Martínez et al., 2020a [37]	Cross-sectional	1752 derivation cohort 875 validation cohort	NI	PPQ CH	PPQ = NI	Not established	INT-PD ACT-PH
Martínez-Vázquez et al., 2021 [38]	Cross-sectional	899	0 to 12 months postpartum	AdHoc-SC AdHoc-VC AdHoc-OV PPQ	PPQ ≥ 19	Inadequate healthcare or treatment. Break of autonomy	INT-PD ACT-PH
Martínez-Vázquez et al., 2021a [39]	Cross-sectional	1.301	12 to 36 months postpartum	AdHoc-SC AdHoc-VC AdHoc-OV PPQ	PPQ ≥ 19	Verbal, physical, and psycho-affective abuse during care at delivery	INT-PD ACT-P
Janevic et al., 2021 [53]	Cross-sectional	237	0 to 2 months postpartum	AdHoc-SC BSS-R DMS PHQ-2 C-DSM-V	C-DSM-V at least one criterion	Not established	ACT-PH PER-SC
Kountanis et al., 2022 [40]	Longitudinal	112	6 weeks postpartum, 3 months postpartum, 6 months postpartum, and 1 year postpartum	AdHoc-SC AdHoc-OV PPQ CH	PPQ ≥ 13	Not established	PER-SC
Yakupova et al., 2021 [52]	Cross-sectional	611	14 months postpartum	AdHoc-SC AdHoc-OV EPDS CBTS	CBTS = NI	Not established	INT-PD ACT-PH
Steetskamp et al., 2022 [41]	Longitudinal	589 * 278 **	1 day postpartum and 6 months postpartum	AdHoc-SC AdHoc-VC IES-R	IES-R ≥ 0	Unsatisfied expectations during delivery and non-adaptation to reactions of the obstetric teams in complications during delivery	INT-PD ACT-PH
Yakupova et al., 2022 [42]	Cross-sectional	611	0 to 6 months postpartum	AdHoc-SC AdHoc-OV CBTS	CBTS = NI	Not established	INT-PD ACT-PH

* Finished the first assessment; ** completed the final assessment. PTSD: post-traumatic stress disorder. NI: was not exposed in the document. OV: obstetric violence or vulnerability due to healthcare. AdHoc-SC: ad hoc questionnaire to collect sociodemographic data. AdHoc-VC: ad hoc questionnaire for clinical variables. AdHoc-OV: ad hoc questionnaire to identify actions associated with obstetric violence or birth experience. PSS-SR: PTSD Symptom Scale. SF-MB: standardized form about the mode of birth. IES-R: Impact of Event Scale—Revised. TES: Traumatic Event Scale. CH: clinic history. PPQ: Perinatal Post-traumatic Stress Disorder Questionnaire. BSS-R: Birth Satisfaction Scale—Revised. DMS: Discrimination in Medical Settings Scale. PHQ-2: Patient Health Questionnaire. C-DSM-V: DSM-V diagnostic criteria. CBTS: City Birth Trauma Scale. The measurement of OV was classified as follows: INT-PD: medical interventions that are painful or disabling and could be avoided under clinical supervision (episiotomy, amniotomy, Kristeller technique, C-section, instrumental labor, perineal tears, use of the synthetic oxytocin, manual removal of the placenta, and birth injury), ACT-PH: actions of the professional healthcare (mistreatment, discrimination, offensive and coercive communication, disrespect to labor plan and privacy, poor clarity in the information, minimize women´s autonomy, bullying, refusal of pain relief, and deprive women of expressing their emotions or questions, skin-to-skin with infant, breastfeeding in the first hour after delivery and social support); and PER-SC: perception about the satisfaction of healthcare during childbirth based on meeting needs.

**Table 3:** Prevalence and factors associated with obstetric violence in postpartum depression.

Document	Prevalence of PPD	Prevalence of Different Forms of OV	Factors Associated with OV Contributing to PPD Development
Theme Filha et al., 2016 [43]	26.3% after 6 h postpartum, 25.7% at 45 days to 6 months postpartum, and 27.1% at between 6 and 18 months postpartum	Not social support during labor and birth = 24.4%. Not painful labor analgesia = 20.8%. Self-rated bad or very bad care during birth = 2.4%. Self-rated bad or very bad care of the newborn = 1.7%.	Risk factors associated: Not allowed social support during labor (aOR: 1.18 [0.91; 1.54]), allow partner for a short time (aOR: 1.24 [0.98; 1.56]). Poor self-rated care during birth (OR: 2.02 [1.28; 3.20]) and poor self-rated newborn (OR: 2.16 [1.51; 3.10]).
De Souza et al., 2017 [44]	18.4%	Physical violence = 59.0%. Violence by negligence = 51.8%. Verbal violence = 50.3%. Violence from the institution = 26.1%. Violence from the health system = 19.6%.	Risk factors associated: Physical violence (aOR: 5.83 [4.95; 6.87]). Violence by negligence (aOR: 7.66 [6.37; 9.23]). Verbal violence (aOR: 5.93 [5.14; 6.87]). The physical violence was the main risk factor, being modified by women’s age. Protective factors associated: The social support during labor (OR: 0.39 [0.30; 0.34]). The age modified the effect of negligence (aOR: 0.10 [0.03; 0.15]).
Hartmann et al., 2017 [45]	14%	Low support from health team = 10.2%. Poor monitoring for healthcare staff = 2.3%.	Protective factors associated: The support from health team during birth (OR: 0.77 [0.61; 0.96]) and professional monitoring during hospitalization (partial = OR: 0.90 [0.57; 1.43]; continuous = OR: 0.47 [0.29; 0.74]).
Silveira et al., 2019 [46]	30% with depressive symptoms during pregnancy (EPDS ≥ 10). 9.4% with moderate postpartum depression (EPDS ≥ 13). 5.7% with severe postpartum depression (EPDS ≥ 15).	Any disrespect and abuse during childbirth = 18.0%. Verbal abuse = 9.0% Physical mistreatment = 5.0%, Denial of care = 6.0%. Undesirable procedures = 6.0%.	Risk factors associated: The verbal abuse was for moderate PPD (aOR: 1.58 [1.06; 2.33]), 1 type of abuse to severe PPD (aOR: 1.56 [1.07; 2.27]), being higher for physical abuse (aOR: 2.26 [1.26; 4.08]). The verbal abuse to severe PPD (aOR: 1.69 [1.06; 2.70]). Experience 3 or more types of disrespect with moderate PPD (aOR: 2.90 [1.30; 6.48]) and with severe PPD (aOR: 3.86 [1.58; 9.42]).
Leite et al., 2020 [47]	26.3% In women with vaginal birth, being in the public sector 27.0% and in the private sector 16.0%. 23.8% of women with C-section, in the private sector, 16.0%, and in the public sector, 28.7%.	Vaginal: in the public sector = 30.3% vs. in the private sector = 45.2%. C-section: in the public sector = 36.2% vs. in the private sector = 25.3%.	Risk factors associated: The disrespect was the major factor in the public (vaginal: β = 0.22; C-section: β = 0.26) and private health systems (vaginal: β = 0.25; C-section: β = 0.219). In women with vaginal labor, pressure during labor (β = 0.13) was associated with disrespect. In women with C-section, disrespect was associated with maternal hospitalization (β = 0.153) and perform a C-section when the desire was vaginal birth (β = 0.125). In the private sector, disrespect was associated with does not follow the women’s desire for birth (vaginal: β = 0.448; C-section: β = 0.144). Protective factors associated: The social support (β = −0.228) and good practices (β = −0.104). The disrespect and abuse were lower when the women were accompanied (β = −0.143).
Oelhafen et al., 2021 [48]	27%	Informal coercion (intimidation or manipulation) = 26.7% Pressured to consent (inadequate information, insufficient time to make a decision, and disrespect at the opposition) = 16.3%. Misinformation of the professional healthcare provider = 9.5%.	Risk factors associated: Experiencing informal coercion (RR: 1.35 [1.19; 1.54]). Women had risk of informal coercion in urban areas (RR: 1.16 [1.09; 1.23]), preferred to have autonomy in decisions (RR: 1.15 [1.10; 1.21]), high-risk pregnancy (RR: 1.25 [1.10; 1.41]), not give birth where they had initially planned (RR: 1.47 [1.25; 1.73]).
Singh et al., 2021 [49]	33.7%	Disrespect treatment = 54.9%. not explanation about treatment = 40.9%. Dissatisfied with the newborn care = 46.2%.	Risk factors associated: The dissatisfaction with newborn care (OR: 2.91 [1.91; 4.44]).
Janevic et al., 2021 [53]	13 women with 69.2% satisfaction and 35.7% with at least one discriminatory event in medical care during childbirth.	Women who delivered vaginally: Low birth satisfaction = 41.4%. At least 1 discriminatory event in medical care during childbirth = 15.0%. Women who delivered by C-section: Low birth satisfaction = 73.1% At least 1 discriminatory event in medical care during childbirth = 40.4%.	Protective factors associated: The birth satisfaction (aOR 0.1 [0.03; 0.70]).
Paiz et al., 2022 [50]	28.6%	Not feeling comfortable asking questions = 15.1%. Not understanding received information = 12.5%. Not allowing newborn skin-to-skin contact = 32.0%. Not feeling welcome in the birth unit = 21.7%. Not feeling safe in the birth unit = 25.9%. Lack of privacy during birth = 16.1%.	Risk factors associated: Mistreatment during the birth (RR: 1.58 [1.09; 2.29]).
Yakupova et al., 2021 [52]	45.7%	At least 1 case of obstetric violence = 22.6%. Verbal aggression = 11.3%. Medical interventions without consent = 6.2%. Threats and accusations = 4.4%. Pain relief denial = 3.1%. Use of Kristeller technique = 3.1%. Ignoring the needs of the woman = 2.9%. Not social support = 42.1%. Amniotomy = 45.7%.	Risk factors associated: Experienced obstetric violence (β = 2.08 [1.39; 2.78]).
Martinez-Vázquez et al., 2022 [51]	25.4%	Verbal violence = 24.4%. Physical violence = 53.5%. Psycho-affective violence = 35.2%. Induction of labor = 40.2%. Instrumental birth = 19.4%. Emergency C-section = 14.2%. Several perineal tear = 3.3%. Not allowing newborn skin-to-skin contact = 20.7%. Not partner support during childbirth = 1.9%.	Risk factors associated: Verbal violence (aOR: 2.02 [1.35; 3.02]), and psycho-affective violence (aOR: 2.65 [1.79; 3.93]).

PPD: postpartum depression; OV: obstetric violence or vulnerability due to healthcare; OR: odd ratio; aOR: adjusted odd ratio; β: standardized coefficient extracted by the regression models; RR: risk ratios. All regression coefficients were expressed with a 95% confidence interval.

**Table 4:** Prevalence and factors associated with obstetric violence in post-traumatic stress disorder.

Document	Prevalence of PTSD	Prevalence of Different Forms of OV	Factors Associated with OV Contributing to PTSD Development
Haagen et al., 2015 [33]	0.57% at 3 months postpartum and 0.35% at 10 months postpartum	Vaginal labor = 12.1%. Vacuum extraction = 8.1%.	Risk factors associated: Poor perinatal information by staff (β = 0.16) and mode of delivery (β = 0.15). The indirect effect of negative emotion was somatic (β = 0.14) and psychological dissociation (β = 0.041).
De Schepper et al., 2016 [34]	0.3% (TES) y 5.7% (IES-R) at first week postpartum. 0% (TES) and 4% (IES-R) at 6 weeks postpartum	Unplanned C-section = 39.0%. Induction of labor = 33.0%. Episiotomy = 41.4%. Instrumental delivery = 10.9%.	Protective factors associated: In the first week postpartum, the women´s locus of control during the birth (β = −0.274). At 6 weeks postpartum, the perception of women about the midwife having control during labor and birth (β = −0.25) and the possibility of asking questions during labor (β = −0.153). Women with spontaneous labor had 88% reduction in developing PTSD.
Hernández- Martínez et al., 2019 [35]	10.6%	Unfollow the birth plan = 79.4%. Kristeller technique = 30.8%. Episiotomy = 36.4%. Perineal tears = 4.2%. Not allowing newborn skin-to-skin contact = 32.4%. Not using analgesia = 22.5%. Not using anesthesia = 96.3%.	Risk factors associated: Instrumental delivery (aOR: 2.50 [1.70; 3.69]), C-section (aOR: 3.79 [2.43; 5.92]), Kristeller technique, (aOR: 1.48 [1.12; 1.97]), perineal tears (aOR: 2.77 [1.71; 4.49]), and use anesthesia (aOR: 1.92 [1.21; 3.05]). Protective factors associated: Respected birth plan (aOR: 0.52 [0.34; 0.80]), using epidural analgesia (aOR: 0.64 [0.44; 0.92]) and skin-to-skin contact (aOR: 0.37 [0.28; 0.50]).
Hernández-Martínez et al., 2020 [36]	7.2% in women between 1 and 3 years postpartum. 8.1% in women between 4 and 5 years postpartum 5.9%.	Unfollow the birth plan = 18.9%. Induced birth = 32.9%. Fundal pressure = 31.0%. Instrumental delivery = 17.7%. C-section = 24.9%. Episiotomy = 40.0%. Perineal tears = 4.4%. Not allowing newborn skin-to-skin contact = 35.5%. Not using analgesia = 22.9%. Not using anesthesia = 96.4%.	Risk factors associated: Instrumental delivery (aOR: 3.32 [1.73; 3.39]), C-section (aOR: 4.80 [2.51; 9.15]), fundal pressure (aOR: 1.72 [1.08; 2.74]) and perineal tears (aOR: 2.73 [1.27; 5.86]). Protective factors associated: Respected birth plan (aOR: 0.44 [0.19; 0.99]), use of epidural (aOR: 0.44 [0.24; 0.80]), and skin-to-skin contact at birth (aOR: 0.33 [0.20; 0.55]).
Hernández-Martínez et al., 2020a [37]	14.2%	Labor induction = 40.6%. Instrumental delivery = 18.6%. Episiotomy = 28.9%. Perineal tear = 3.9%. Not allowing newborn skin-to-skin contact = 23.6%. Disrespect birth plan = 14.3%.	Risk factors associated: Several perineal tear (OR: 2.21 [1.17; 4.19]) and instrumental delivery (OR: 1.62 [1.10–2.41]). Protective factors associated: Perception of respect from healthcare professionals (OR: 0.42 [0.37; 0.48]) and allow skin-to-skin (OR: 0.65 [0.45–0.96]).
Martinez-Vázquez et al., 2021 [38]	12.7%	Verbal violence = 32.3%. Physical violence = 17.6%. Psycho-affective violence = 27.0%. Disrespect birth plan = 39.2%. Induction of labor = 16.5%. Instrumental delivery = 11.8%. Emergency C-section = 33.1%. Episiotomy = 9.5%. Perineal tear = 16.5%.	Risk factors associated: Disrespect birth plan (aOR: 2.85 [1.56; 5.21]), scheduled C-section (aOR: 2.53 [1.02; 2.26]), emergency C-section (aOR: 3.58 [1.83; 6.99]), verbal violence (aOR: 5.07 [2.98; 8.63]) and psycho-affective violence (aOR: 2.61 [1.45; 4.67]).
Martínez-Vazquez et al., 2021a [39]	13.1%	Verbal violence = 31.2%. Physical violence = 16.9%. Psycho-affective violence = 25.4%. Disrespect birth plan = 31.3%. Induction of labor = 16.0%. Instrumental delivery = 17.0%. Emergency C-section = 24.9%. Episiotomy = 15.4%. Perineal tear = 27.5%. Not allowing newborn skin-to-skin contact = 23.8%. Not breastfeeding 1 h after childbearing = 22.4%. Feeling disrespect by health professionals = 65.5%.	Risk factors associated: Disrespect birth plan (aOR: 1.89 [1.21; 2.94]), verbal (aOR: 3.73 [2.52; 5.53]), and physical violence (aOR: 3.98 [2.48; 6.39]). Induction of labor (OR: 1.50 [1.09; 2.06]), instrumental delivery (OR: 2.20 [1.42; 3.39]), emergency C-section (OR: 3.57 [2.41; 5.28]), and several perineal tears (OR: 2.26 [1.18; 4.30]).
Janevic et al., 2021 [53]	100% reported low birth satisfaction, and 63.6% reported at least 1 discriminatory event in medical care.	Women who delivered vaginally: Low birth satisfaction = 41.4%. At least 1 discriminatory event in medical care during childbirth = 15.0%. Women who delivered by C-section: Low birth satisfaction = 73.1% At least 1 discriminatory event in medical care during childbirth = 40.4%.	Risk factors associated: Experiencing any discrimination during childbirth (aOR: 3.2 [1.1; 9.4]).
Kountanis et al., 2022 [40]	20.7% at 6 weeks, 18.7% at 3 months, 17% at 6 months, and 24.5% at 12 months postpartum.	Perceived negative communication with the medical staff = 26.4%.	Protective factors associated: Positive perception of communication with the medical staff at 6 months (OR: 0.29) and 12 months postpartum (OR: 0.20).
Yakupova et al., 2021 [52]	15.1%	Experienced at least 1 case of obstetric violence = 22.6%. Verbal aggression = 11.3%. Medical interventions without consent = 6.2%. Threats and accusations = 4.4%. Pain relief denial = 3.1%. Use of Kristeller technique = 3.1%. Ignoring the needs of the woman = 2.9%. Not social support = 42.1%. Episiotomy = 19%. Amniotomy = 45.7%. C-section = 22.6%.	Risk factors associated: Experienced obstetric violence (β = 5.09 [3.81; 6.38]).
Steetskamp et al., 2022 [41]	2.9%	Not social support during labor = 35.0%. Birth injury = 55.0%. C-section = 17.0%. Vacuum extraction = 5.9%.	Risk factors associated: The mode of delivery (C-section: RR = 0.103). Protective factors associated: The assistant companion (social support: RR = −0.369).
Yakupova et al., 2022 [42]	15.0%	Bullying = 11.3%. Intervention without consent = 6.2%, Threats and accusations = 4.4%. Use of Kristeller technique = 3.1%. Denial of pain relief = 3.1%. Amniotomy = 45.7%. Episiotomy = 19.0%. C-section = 22.6%.	Risk factors associated: Increased medical interventions (β = 1.03 [0.23; 1.84]) and perception of obstetric violence (β = 5.08 [3.80; 6.37]).

PTSD: post-traumatic stress disorder; OV: obstetric violence or vulnerability due to healthcare; OR: odd ratio; aOR: adjusted odd ratio; β: standardized coefficient extracted by the regression models; RR: risk ratios. All regression coefficients were expressed with a 95% confidence interval.

## Data Availability

No new data were created or analyzed in this study.

## Public Involvement Statement:

No public involvement in any aspect of this research.

## Guidelines and Standards Statement:

This manuscript was drafted against the PRISMA 2020 statement preferred reporting items for systematic reviews and meta-analysis research [25].
